# Spatiotemporal trends and the change detection of the yearly eco-environmental quality in the Yellow River Basin, China

**DOI:** 10.1038/s41598-026-48716-8

**Published:** 2026-04-20

**Authors:** Hui Ye, Jiangbao Xia, Xiao Wang, Shuo Li, Qiqi Cao, Haidong Xu, Xiaodong Li

**Affiliations:** 1 Shandong Key Laboratory of Eco-Environmental Science for the Yellow River Delta, Shandong University of Aeronautics, Binzhou, 256603 China; 2 Institute of Restoration Ecology, School of Chemical and Environmental Engineering, China University of Mining and Technology (Beijing), Beijing, 100083 China

**Keywords:** Yellow River Basin (YRB), functional zones, the change detection, the yearly Eco-Environmental Quality, land cover type conversion, Ecology, Ecology, Environmental sciences

## Abstract

As a critical ecological security and economic corridor in China, monitoring the ecological quality of the Yellow River Basin (YRB) is essential for China’s ecological protection and sustainable economic development. In this study, we constructed a modified Remote Sensing Ecological Index (MRSEI) to investigate the spatiotemporal dynamics of ecological quality in the YRB from 2001 to 2023. Meanwhile, we incorporated the Aridity Index (AI) and the temperature (T). Furthermore, Multiple Linear Regression and Random Forest models were employed to quantify the contributions of moisture (NDMI), urbanization (NDBI), and productivity (NPP) to ecological variance. Finally, land cover type conversion was employed to interpret ecological variations detected by the MRSEI dynamic ratio. The primary results are as follows: (1) Ecological quality in the middle reaches of the YRB significantly improved from 2001 to 2010, remaining relatively stable from 2011 to 2023. (2) No significant correlation was observed between temperature (T) and MRSEI, but significant correlations between Aridity Index (AI) and MRSEI were observed during 2001–2010 in the Windbreak and Sand Fixation Functional Zone (WSFZ, r_2001−2010_=0.520 ± 0.225, *p* < 0.01) and Soil Conservation Functional Zone (SCFZ, r_2001−2010_=0.467 ± 0.350, *p* < 0.01). (3) The dominant land cover conversion types include barred land-to-cropland conversion (3640.44 km^2^) and cropland/barren land-to-grassland conversion (3212.19 km^2^; 1507.25 km^2^) in regions where ecological conditions improved from 2001 to 2023. (4) Form 2001–2023, NDMI (Surface Moisture), contributes 52%-56% to ecological dynamics, while the water cycle budget exerts a governing influence on basin-wide ecological quality. This study offers critical insights for promoting ecological protection and sustainable development in the functional zones of the YRB.

## Introduction

As both a critical ecological security barrier and an economic corridor in China, the Yellow River Basin (abbreviated as YRB) plays a pivotal role in supporting the sustainable development of the nation’s natural environment and economy^1,2^. Due to its extensive span across diverse geomorphological zones, the YRB confronts multiple ecological challenges^3,4^, including persistent drought, water scarcity, and severe soil erosion in the upper and middle reaches^5^; meanwhile, vegetation degradation and biodiversity loss in the lower reaches are further exacerbated by rapid development of urbanization and industrialization^6^, which has significantly contributed to China’s sustained economic growth^7,8^. Therefore, accurately assessing the eco-environmental quality of the YRB is of great importance for advancing its ecological conservation and sustainable development.

Methods such as the Analytic Hierarchy Process (AHP), Weights-of-Evidence and “Pressure-State-Response” (PSR) have been widely employed assess the terrestrial ecosystems quality^9–11^; however, these approaches are constrained by limitations in indicator data collection^12^. So far, remote sensing technology has been extensively applied^13,14^ due to its advantages of its broad spatial coverage and high temporal resolution. A variety of single ecological indices have been utilized to interpret specifical ecological attributes of particular environmental components^15,16^, including the vegetation index (Normal Difference Vegetation Index, NDVI), the water index (Normal Difference Water Index, NDWI) and the soil index (Normal Difference Bare Soil Index, NDBSI). However, merely reliance on a single ecological indicator fails to capture the multi-dimensional nature of ecosystem quality, as it overlooks critical factors like climate variability and anthropogenic disturbances^17^.

The Remote Sensing Ecological Index (RSEI), which integrates vegetation information, wetness component, land surface temperature (LST) and soil information^18^, has been widely adopted as a principal tool for assessing urban ecological quality^19,20^. However, its application has certain limitations, including the lack of scenario diversity, uncertainty, and temporal inconsistency^21^. To overcome these limitations, scholars have proposed various improvements to the RSEI through two methodological approaches. On the one hand, additional indicators were introduced to more accurately capture the ecological characteristics of the study area^12,22^. For example, an integrated salinity index (ISI) was introduced, tailored to the salinized soil conditions prevalent in agricultural regions, into the traditional RSEI model, providing a rapid and effective means for monitoring ecological quality in agricultural regions affected by soil salinization^23^. On the other hand, alternative methods were explored to calculate RSEI instead of using Principal Component Analysis (PCA). Chen et al., for instance, employed the entropy weighting method, incorporating spatiotemporal features and seasonal variations, to develop an improved remote sensing ecological index (IRSEI) that enhanced data utility compared to conventional approaches^24^. While previous studies focus on static indices, this study employs a dynamic ratio detection algorithm to isolate interannual shifts, subsequently validating these shifts through both linear (MLR) and non-linear (Random Forest) importance assessments.

To comprehensively evaluate the ecological quality of the YRB, this paper aims to: (1) utilize the modified normalized difference water index (MNDWI) and Albedo to replace the original moisture and dryness indicators to construct the modified RSEI; (2) incorporate the Aridity Index (AI) and the temperature (T) to analyze their correlation with MRSEI; (3) calculate the MRSEI dynamic ratio by constructing an ecological change detection algorithm to quantify the extent of ecological quality improvement; (4) quantitatively evaluate the contribution rates of individual variables (including natural factors and anthropogenic factors) to the RSEI, based on linear (Multiple Linear Regression) and non-linear (Random Forest) models.

## Study area and datasets

### Study area

**Fig. 1:**
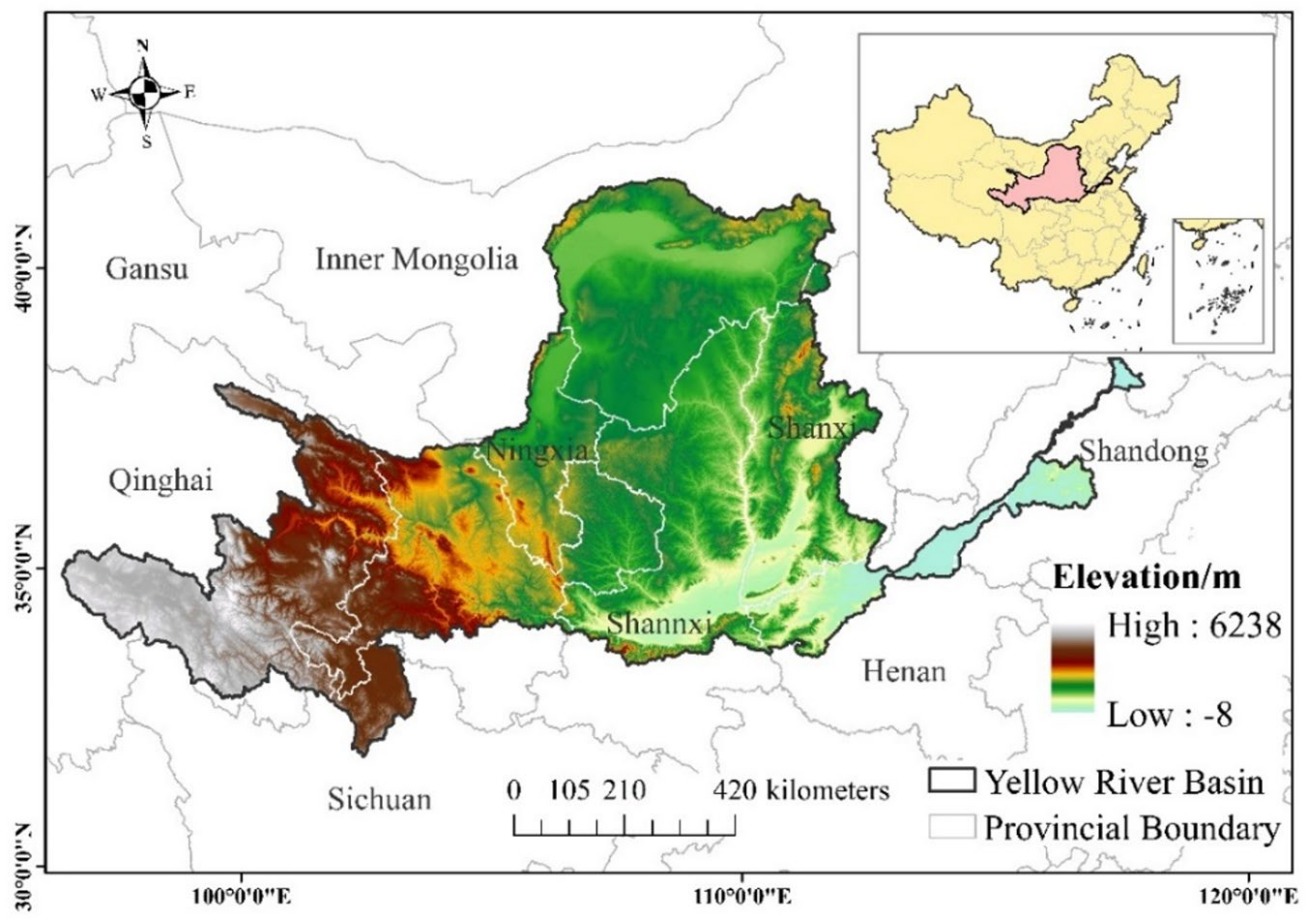
Location of the Yellow River Basin.

The YRB extends across the western, northern and central parts of China (95°53′E-119°05′E, 32°10′N-41°50′N), originating in the Bayan Har Mountains on the Qinghai-Tibet Plateau and flowing eastward into the Bohai Sea (Fig. 1). Its drainage area encompasses nine provincial-level regions - Qinghai, Sichuan, Gansu, Ningxia, Inner Mongolia, Shaanxi, Shanxi, Henan, and Shandong - and covers approximately 79,500 km^2^^25^. And the YRB exhibits diverse climate types, with a gradual transition from the dry and cold western regions to the warmer and more humid eastern areas^25–27^. Known as China’s second-largest river basin, the YRB supplies fresh water to 8.7% of China’s population^26,28^. In recent years, it has experienced rapid economic development, emerging as a crucial driver supporting China’s overall development^29^.

### Data sources and pre-processing

Reflective spectral bands 1–7 of MOD09A1 production, after undergoing standard preprocessing followed by additional processing steps (clipping, projection transformation, outlier removal, and water body masking), were used to compute the RSEI. The Aridity Index (AI) was calculated from precipitation data^30^ and potential evapotranspiration data^31^, both with a spatial resolution of 1000 m. Temperature data (also at a 1000 m resolution), including the T-mean of the growing season, were obtained from Geographic Data Sharing Infrastructure, a global resources data cloud (www.gis5g.com). And the CLCD dataset^32^ was provided by National Cryosphere Desert Data Center (http://www.ncdc.ac.cn) and resampled to a 250 m spatial resolution (Table 1).

**Table 1:** Data sources.

No.	Data type	Time period	Data Product/Name	Source
1	Reflective spectral bands 1–7	2001–2023	MOD09A1	GEE
2	Air Temperature (TEM)	2001–2023	China 1 km Monthly Mean Temperature Dataset	Earth Resource Data Cloud
3	Precipitation (PRE)	2001–2023	China 1 km Annual Precipitation Dataset	Earth Resource Data Cloud
4	Land Surface Temperature (LST)	2001–2023	MOD11A2	GEE
5	Shortwave Albedo	2001–2023	MCD43A3	GEE
6	LUCC	2000, 2005, 2010, 2015, 2020	China’s Multi-Period Land Use Land Cover Remote Sensing Monitoring Dataset	National Cryosphere Desert Data Center
7	DEM	2009	ASTER GDEM	Earth Engine Data Catalog
8	Nighttime Light (NLS)	2001–2023	Extended VIIRS-Like Nighttime Light Dataset	Earth Resource Data Cloud
9	Population Density (POP)	2001–2023	LandScan Global Dataset	Earth Resource Data Cloud
10	Actual Evapotranspiration (ET)	2001–2023	MOD16A2GF	GEE
11	Net Solar Radiation (SAR)	2001–2023	MYD15A2H	GEE
12	Daily Meteorological Data	2001–2023	ERA5_LAND/DAILY_AGGR	GEE

All other datasets were retrieved from the Google Earth Engine (GEE) platform, with data processing and collection conducted via Python-based programming.

## Methods

**Fig. 2:**
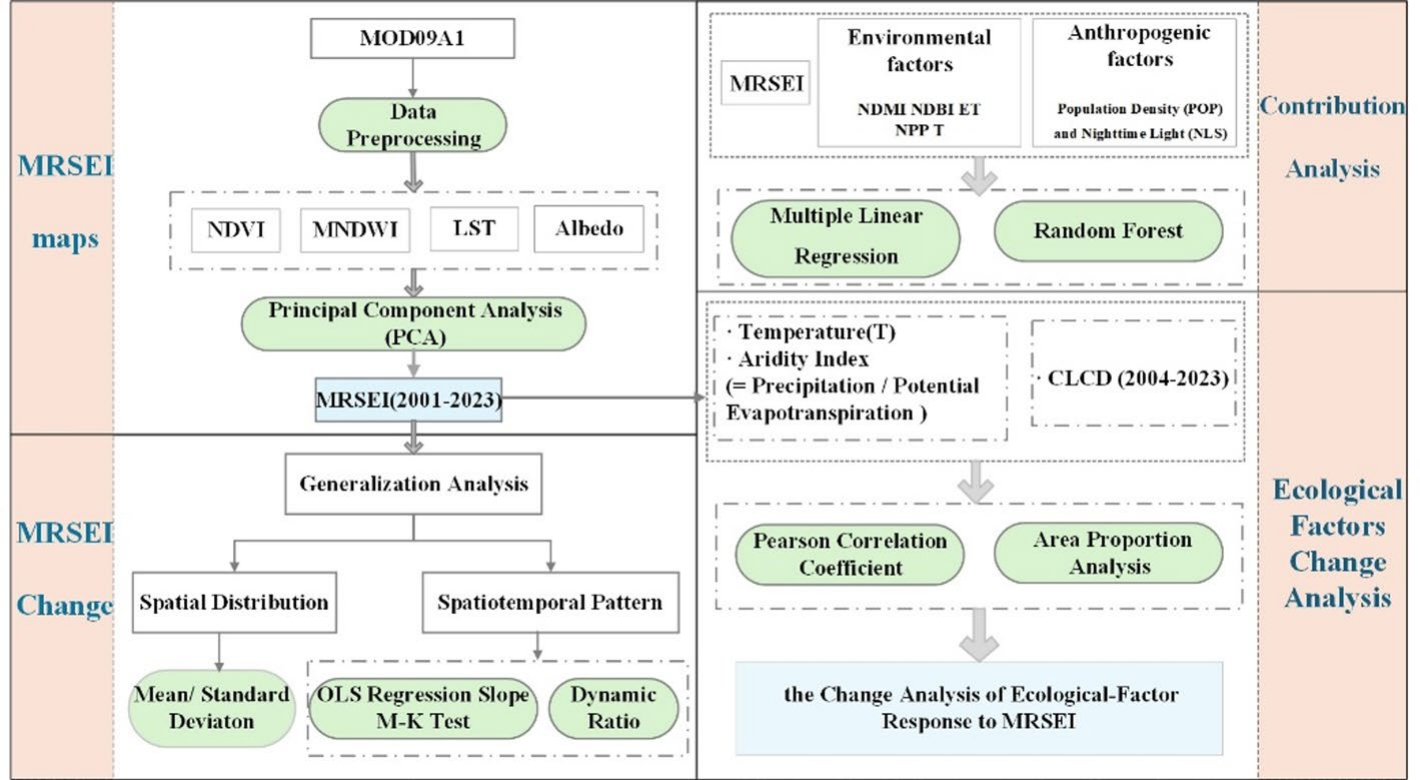
Technology flowchart.

This study includes MRSEI construction, spatiotemporal analysis of ecological quality in the YRB, relationship between MRSEI and temperature (T) and aridity index (AI) (Fig. 2).

### MRSEI index calculation

To better reflect the ecological quality conditions of the YRB, this study replaced WET and NDSI with MNDWI and Albedo in the construction of the MRSEI model (Table 2). Each indicator was standardized to eliminate scale effects and ensure comparability in weight assignment^24^.

**Table 2:** Formulas of the four indicators.

Indicators	Calculation formula
Greenness	$\:NDVI=\frac{{\rho\:}_{NIR}-{\rho\:}_{Red}}{{\rho\:}_{NIR}+{\rho\:}_{Red}}$
Wetness	$\:MNDWI=\frac{{\rho\:}_{Green}-{\rho\:}_{SWIR1}}{{\rho\:}_{Green}+{\rho\:}_{SWIR1}}$
Heat	$\:LST=\frac{T}{[1+\left(\frac{\lambda\:T}{\rho\:}\right)*\mathrm{ln}\epsilon\:]}$
Dryness	$\:Albedo=0.356{\rho\:}_{Blue}+0.130{\rho\:}_{Red}+0.373{\rho\:}_{NIR}+0.085{\rho\:}_{SWIR1}+0.072{\rho\:}_{SWIR2}-0.0018$

Notes: $\:{\rho\:}_{Blue}$, $\:{\rho\:}_{Green}$, $\:{\rho\:}_{Red}$, $\:{\rho\:}_{NIR}$, $\:{\rho\:}_{SWIR1}$, and $\:{\rho\:}_{SWIR2}$ represent the reflectance values of the blue, green, red, near-infrared, shortwave infrared 1, and shortwave infrared 2 bands of the remote sensing imagery, respectively.

The first principal component (PCA1), extracted through principal component analysis (PCA), was selected to construct MRSEI, as it captures over 90% of the total variance in the dataset^33^. The MRSEI was formulated as shown in Formula (1) and normalized as shown in Formula (2).

1 $$\:M{RSEI}_{0}=1-\left\{PCA1\left[f\left(NDVI,MNDWI,LST,Albedo\right)\right]\right\}$$

2 $$\:MRSEI=\frac{M{RSEI}_{0}-{MRSEI}_{0Min}}{M{RSEI}_{0Max}-{MRSEI}_{0Min}}$$

where MRSEI_0_ is the MRSEI in a certain year, MRSEI_0Max_ is the maximum value of the MRSEI and MRSEI_0Min_ is the minimum value of the MRSEI.

### The change detection model based on the yearly MRSEI

A detection model for quantifying interannual variations and assessing terrestrial eco-environmental quality, based on multi-temporal dynamic ratios, is here proposed. This algorithm aims to quantify the interannual change (improvement/degradation) levels of terrestrial eco-environmental quality. Firstly, during the observation period, the multi-temporal dynamic ratio of ecological changes is calculated. Secondly, though progressively applying different time intervals (from small to large), the functional relationship between the ecological quality dynamic ratio and corresponding observation time spans (Δt, 2Δt, 3Δt, …, hΔt, where h + 1 years represent the total observation duration and Δt denotes the base time interval) is constructed in combination with the semi-variogram theory, and the mean dynamic ratio under the same time interval is obtained. Finally, through the extremum detection of the mean dynamic ratios across different time intervals, the change detection (maximum or minimum value) of the dynamic ratio is quantified to measure the interannual variation of terrestrial ecological quality over the observation period, generating a multi-temporal dynamic ratio product dataset that characterizes ecosystem changes.

3 $$\:{S}_{h\varDelta\:t}=\frac{{\Sigma\:}\left(\frac{{X}_{\left(i\:+h\varDelta\:t\right)}-{X}_{i}}{{X}_{i}}\right)}{{N}_{h\varDelta\:t}}$$

4 $$\:{S}_{max}=MAX\left({S}_{1\varDelta\:t\:},{S}_{2\varDelta\:t\:},{S}_{3\varDelta\:t\:},\dots\:\dots\:{S}_{H\varDelta\:t\:}\:\right)\:\left({k}_{xi}>0\right)$$

5 $$\:{S}_{min}=MIN\left({S}_{1\varDelta\:t\:},{S}_{2\varDelta\:t\:},{S}_{3\varDelta\:t\:},\dots\:\dots\:{S}_{H\varDelta\:t\:}\:\right)\:\left({k}_{xi}<0\right)$$

Here, Δ*t* denotes the time span, defined as the temporal distance between one observation and another; *X*_(*i*)_ represents the i-th observation value; *N* is the number of observation values within the *h*Δ*t* time interval; and *S*_*h*Δ*t*​_ stands for the mean dynamic ratio under the Δ*t* time interval. The dynamic ratio (*S*_max_​ or *S*_min_​) can effectively determine the range of ecological changes, identify the direction of ecological change, and quantitatively assess the interannual significance of ecological variations.

### Aridity index

According to the definition introduced by the United Nations Environment Program (UNEP,1993), the Aridity Index (AI) is calculated as the ratio of annual precipitation (P) to potential evapotranspiration (PET)^34^:

6 $$\:AI=\frac{P}{PET}$$

The aridity index classification follows the criteria outlined in Table 3.

**Table 3:** Climate types corresponding to the AI defined by UNEP (1993).

AI	Climate type
[0.05, 0.20)	Arid
[0.20, 0.50)	Semi-arid
[0.50, 0.65)	Dry sub-humid
[0.65, 0.80)	Semi-humid
[0.80, 1.0)	Humid
[1.0, 2.0)	Very humid

### Contribution analysis of ecological factors to RSEI

To evaluate the impact of various ecological factors on the Remote Sensing Ecological Index (RSEI), this study employs a dual-modeling approach using both linear regression and Random Forest algorithms.

#### Linear modeling framework

Linear models, including Generalized Linear Mixed Models (GLMMs), serve as the primary framework for quantifying the direct, additive contributions of environmental variables^35^. These models are particularly effective at accounting for hierarchical data structures, such as spatial or temporal blocking, which are common in ecological datasets. The fundamental mathematical representation is defined as follows:

7 $$\:Y=\:{\beta\:}_{0}\:+\:{\beta\:}_{1}{X}_{1}\:+{\beta\:}_{2}\:{X}_{2}+\dots\:\:+\:{\beta\:}_{n}{X}_{n}+\:\epsilon\:$$

Where, Y represents the ecological response variable (MRSEI). β_n_ denotes the coefficients, which represent the contribution of ecological factor. A higher absolute value of β indicates that the corresponding factor exerts a stronger influence on the ecological outcome. X _n_ is the independent ecological factors.

#### Random forest analysis

While linear models are robust for identifying simple additive relationships, ecological systems are frequently characterized by complex, non-linear interactions that traditional models may fail to capture. Consequently, this study incorporates the Random Forest (RF) model, an ensemble machine learning technique designed to handle non-linear relationships and high-dimensional data^36^.

### Correlation analysis

In this paper, the Pearson Correlation Coefficient was applied to quantify the correlation between temperature utilization efficiency index, aridity index and RSEI. The Pearson Correlation Coefficient is defined as the ratio of the covariance to the product of standard deviations between two variables^37^.

8 $$\:r=\frac{\sum\:_{i=1}^{n}\left({X}_{i}-\mathrm{X}\right)\left({Y}_{i}-\mathrm{Y}\right)}{\sqrt{\sum\:_{i=1}^{n}{\left({X}_{i}-\mathrm{X}\right)}^{2}}\sqrt{\sum\:_{i=1}^{n}{\left({Y}_{i}-\mathrm{Y}\right)}^{2}}}$$

Where, X _i_ and Y _i_ are the individual sample points for the indices being compared. r values range from − 1 to + 1, where values closer to the extremes indicate stronger negative or positive correlations, respectively.

## Results and analysis

### Spatiotemporal evolution of ecological quality

**Fig. 3:**
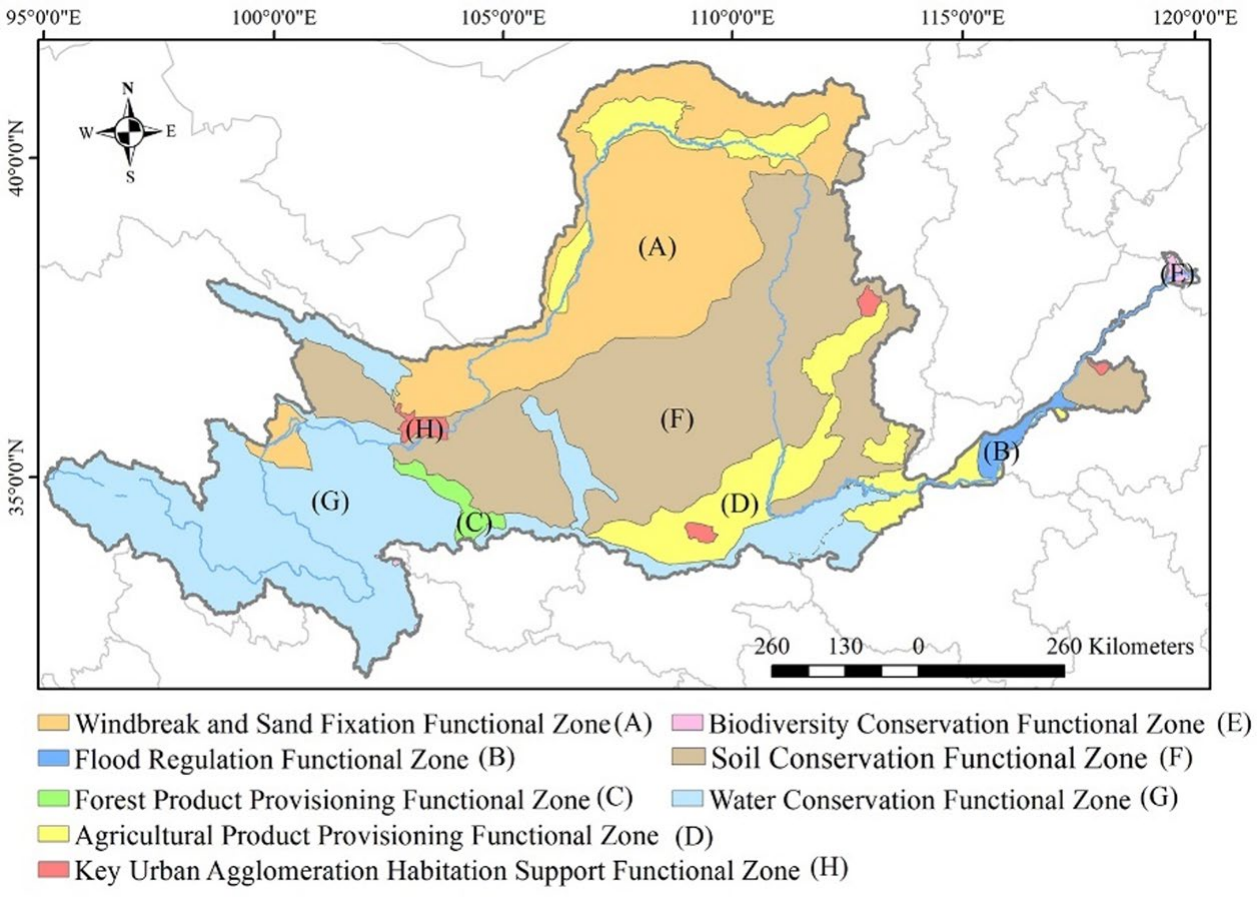
Functional zones classification. Note: Windbreak and Sand Fixation Functional Zone (WSFZ) as A, Flood Regulation Functional Zone (FRFZ) as B, Forest Product Provisioning Functional Zone (FPFZ) as C, Agricultural Product Provisioning Functional Zone (APFZ) as D, Biodiversity Conservation Functional Zone (BCFZ) as E, Soil Conservation Functional Zone (SCFZ) as F, Water Conservation Functional Zone (WCFZ) and Key Urban Agglomeration Habitation Support Functional Zone (KUFZ) are designated as G and H, respectively. And the names of functional zones in this paper are represented by their abbreviations.

In this paper, the MRSEI was categorized into five grades (0.2 intervals): Poor, Fair, Moderate, Good, and Excellent^38^. And then the analysis was carried out at two spatial scales: the Yellow River Basin (YRB; Fig. 1) and its functional subzones (Fig. 3).

**Fig. 4:**
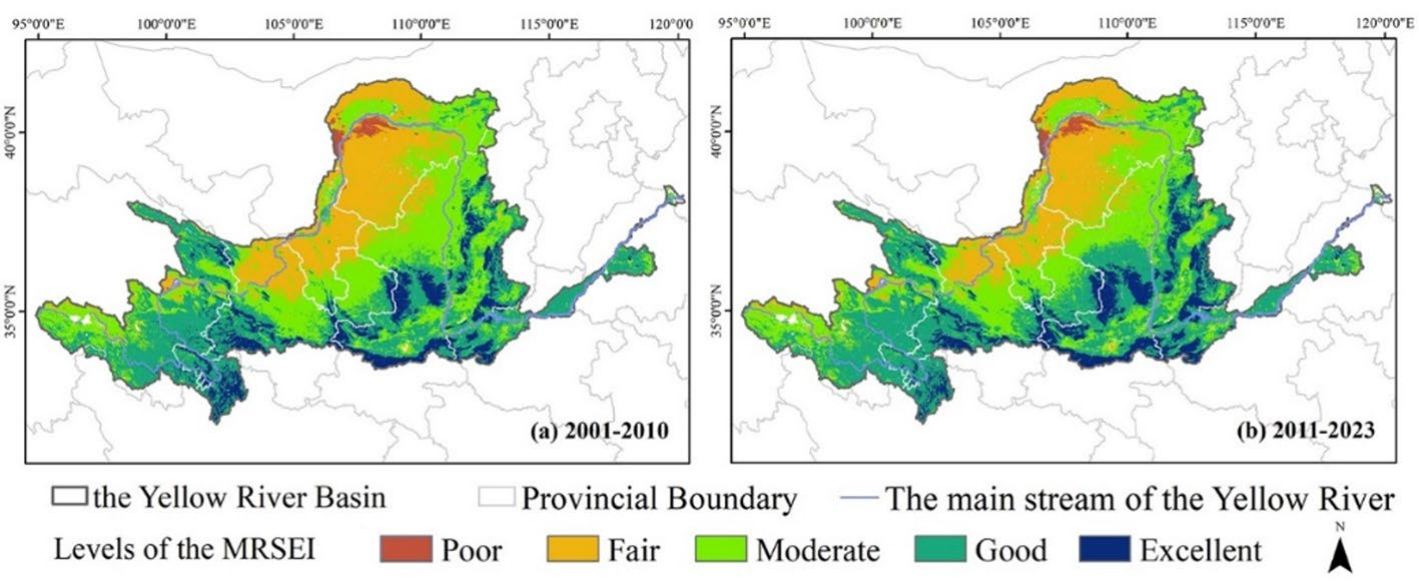
Spatial distribution of mean MRSEI in the Yellow River Basin.

**Fig. 5:**
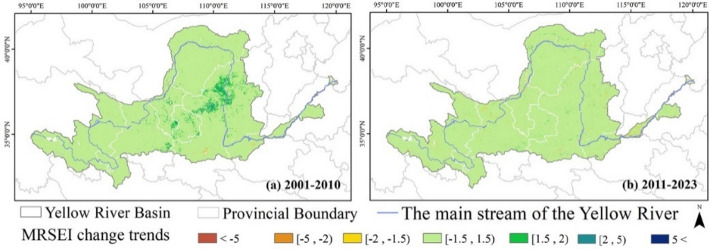
Trends of MRSEI in the Yellow River Basin.

For the YRB, from 2001 to 2010, the MRSEI exhibited a significant increasing trend in the middle reaches (slope *k* > 2, Figs. 4 and 5). Concurrently, areas classified as Good and Excellent levels expanded (45,327 km² and 15,918 km², respectively), while the area of Fair-level regions decreased (50,346 km^2^). From 2011 to 2023, it remained relatively stable (-1.5 < *k* < 1.5), with sporadic areas showing increasing trends (*k* > 2).

**Fig. 6:**
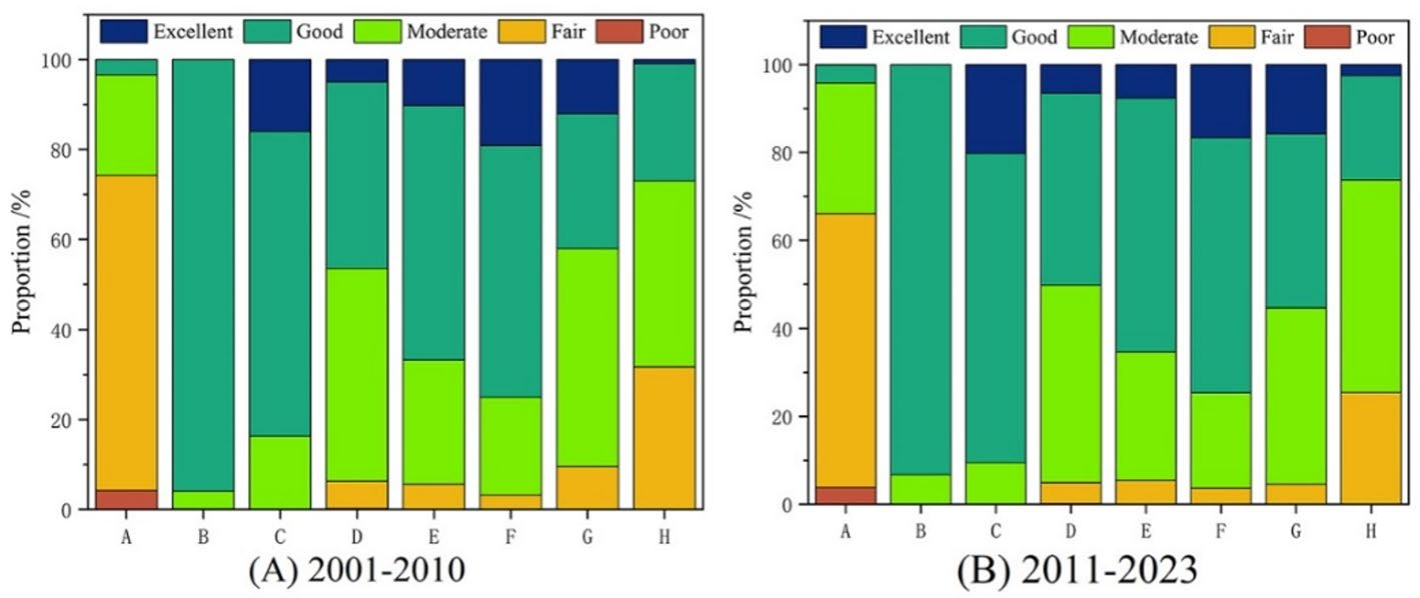
Proportions of areas of the mean MRSEI within functional zones of the YRB.

For different functional zones, the Windbreak and Sand Fixation Zone (WSFZ) had the lowest average MRSEI values (mean ± standard deviation: 0.35 ± 0.11 in 2001–2010; 0.37 ± 0.11 in 2011–2023), with Fair-level areas accounting for 70.00% and 62.28% of the zone, respectively (Fig. 6; Table 5). Conversely, the Flood Regulation Functional Zone (FRFZ) had the highest average MRSEI (0.71 ± 0.05) during 2001–2010, with the Forest Product Provisioning Functional Zone (FPFZ) became the dominant zone with the highest mean MRSEI (0.72 ± 0.09) from 2011 to 2023. The Soil Conservation Functional Zone (SCFZ) exhibited the most significant MRSEI increase (0.04). This suggests that the ecological quality improved in the SCFZ, specifically in the middle reaches of the YRB, from 2001 to 2010.

Overall, the YRB’s ecological quality was classified as Moderate level, with Moderate and Good classes collectively accounting for 77.087% and 80.789%, respectively.

**Table 5:** Mean and standard deviation of MRSEI in different functional zones.

Classification code	Functional zones	2001–2010		2011–2023	
		Mean	Standard deviation	Mean	Standard deviation
A	WSFZ	0.352	0.106	0.374	0.114
B	FRFZ	0.706	0.052	0.687	0.054
C	FPFZ	0.695	0.097	0.722	0.092
D	APFZ	0.588	0.124	0.604	0.126
E	BCFZ	0.643	0.140	0.640	0.131
F	WCFZ	0.685	0.137	0.683	0.139
G	SCFZ	0.586	0.152	0.633	0.145
H	KUFZ	0.495	0.141	0.514	0.138

### Spatiotemporal changes of the temperature (T) and its correlation with MRSEI

**Fig. 7:**
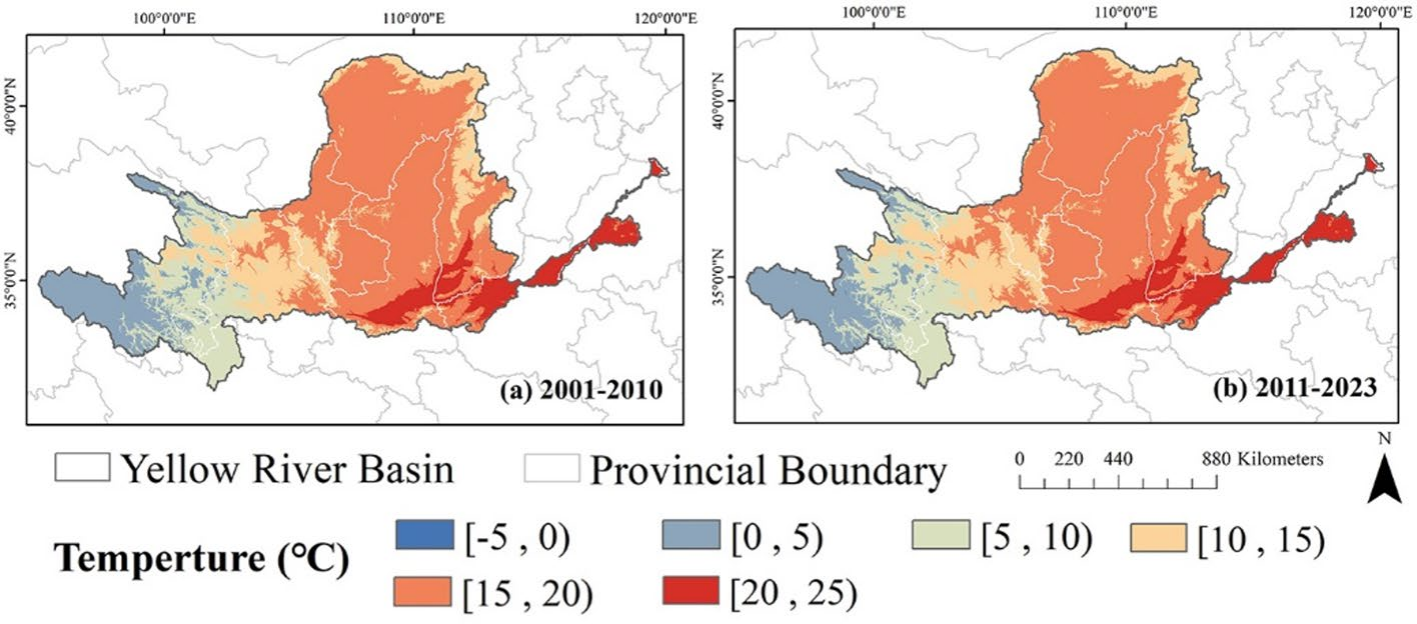
Mean of the T from 2001 to 2010 and from 2011 to 2023.

For the YRB, as shown in Fig. 7, the average T increased successively from the lower, middle to upper reaches, reaching the highest average T in the upper reaches (2001–2010: 0.531 ± 0.116; 2011–2023: 0.542 ± 0.115).

**Fig. 8:**
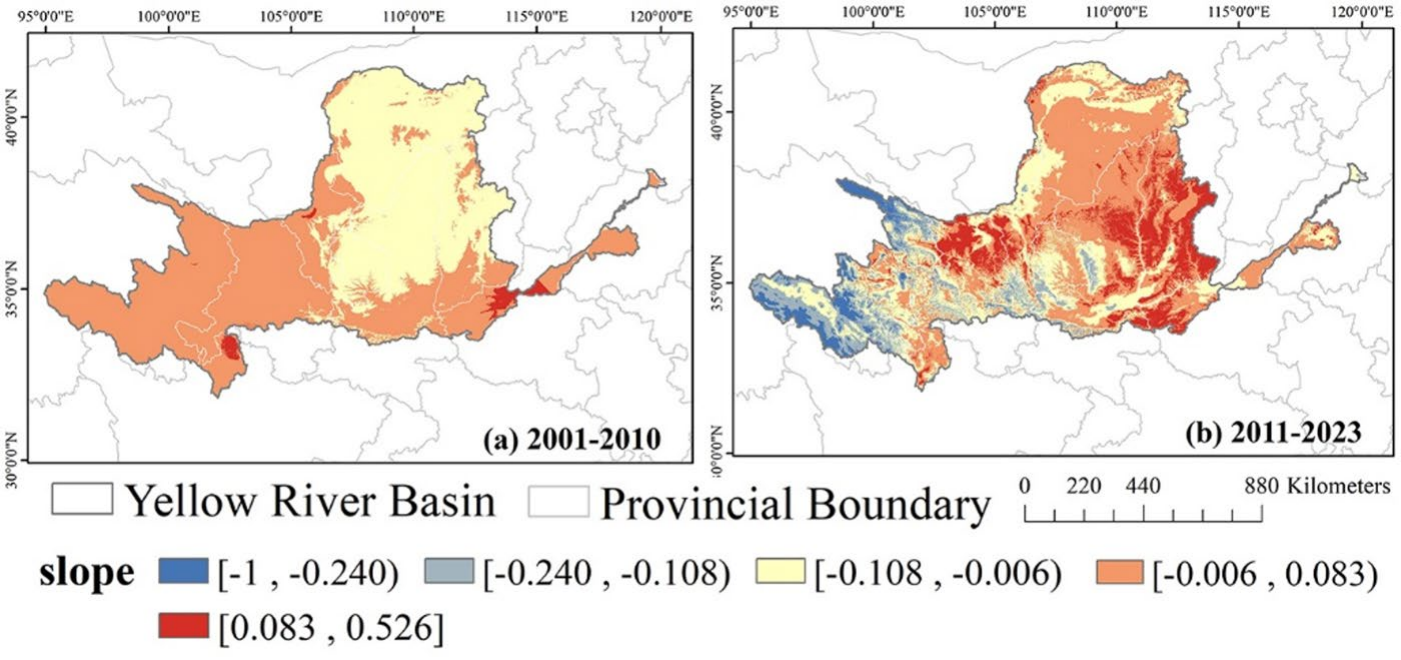
T change trend from 2001 to 2010 and from 2011 to 2023.

For the different functional zones, significant spatial heterogeneity in mean T was observed, with the highest T observed in the FRFZ (2001–2010: 0.535 ± 0.104; 2011–2023: 0.548 ± 0.103). As depicted in Fig. 8, from 2001 to 2010, the T in the Water Conservation Functional Zone (WCFZ) increased at 0.101 ± 0.100 /10a (*p* < 0.05), while the WSFZ and SCFZ decreased at 0.100 ± 0.100/10a and 0.102 ± 0.101/10a, respectively. By contrast, from 2011 to 2023, the T in the WSFZ and SCFZ increased at 0.101 ± 0.101/10a and 0.101 ± 0.102/10a, respectively (*p* < 0.05).

In general, from 2001 to 2010 to 2011–2023, the minimum T was observed in the lower reaches, primarily in the FRFZ. The upper and middle reaches exhibited opposing T trends and concentrated in the WSFZ (upper reach) and SCFZ (middle reach). Subsequently, the correlation between T and RSEI was analyzed by calculating the Pearson Correlation Coefficient across the YRB.

**Fig. 9:**
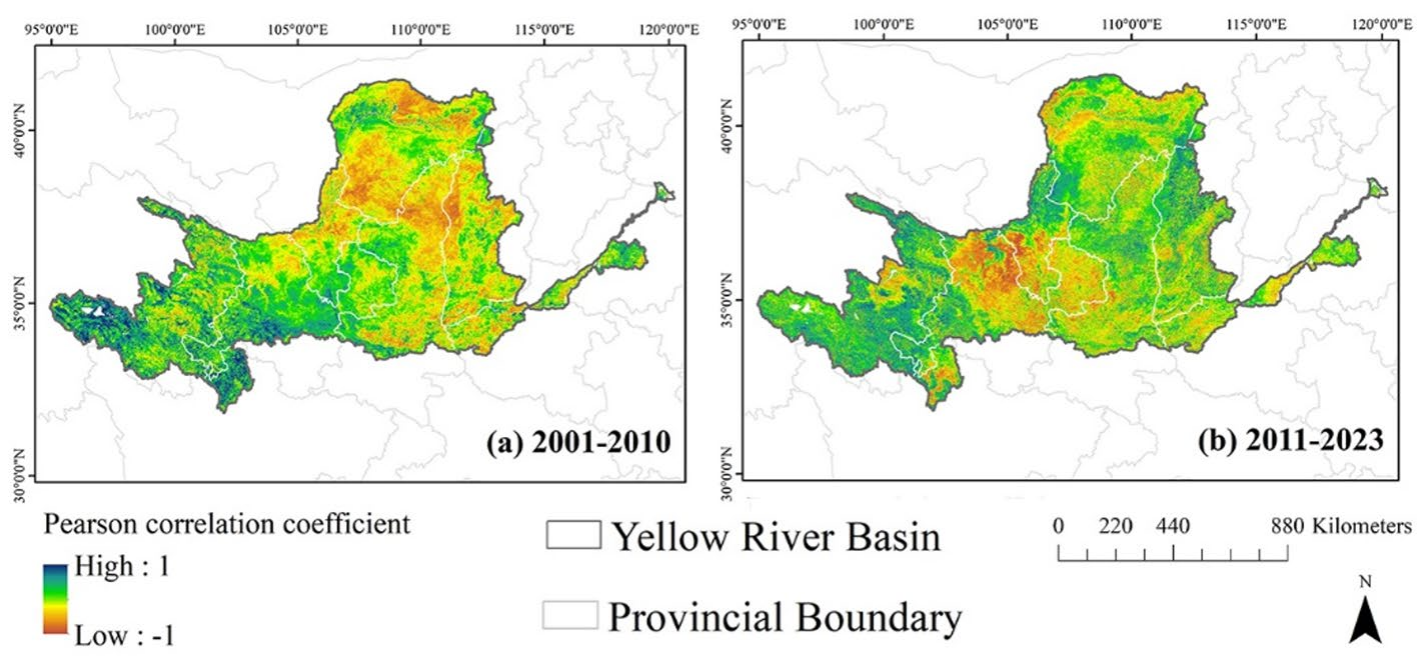
Pearson correlation coefficient between T and MRSEI.

From 2001 to 2010, T and MRSEI were negatively correlated in the northern parts of the upper and middle reaches of the YRB, mainly within the WSFZ (r_2001-2010_=-0.359 ± 0.225, *p* < 0.05) and SCFZ (r_2001-2010_=-0.242 ± 0.248, *p* < 0.05) (Fig. 9; Table 6). The WSFZ exhibited relatively stronger correlation between T and RSEI among all zones (|r_2001-2010_|=0.359).

From 2011 to 2023, a negative correlation was mainly observed in the southwestern parts of the upper and middle reaches of the YRB, still concentrated in the WSFZ and SCFZ. However, the WSFZ (r_2011-2023_=0.015 ± 0.358, *p* < 0.05) and SCFZ (r_2011-2023_=0.015 ± 0.385, *p* < 0.05) exhibited overall positive correlations with MRSEI during this period. Additionally, the WCFZ showed relatively stronger correlation (|r_2011-2023_|=0.221).

These statistical results indicated a generally weak correlation between T and MRSEI, suggesting that T exerts limited influence on ecological quality changes in the YRB.

**Table 6:** Pearson correlation coefficient between T and MRSEI in different functional zones.

Functional zones	2001–2010		2011–2023	
	Mean	Standard deviation	Mean	Standard deviation
WSFZ	-0.359	0.225	0.015	0.258
FRFZ	-0.267	0.243	-0.190	0.220
FPFZ	0.132	0.215	0.088	0.333
APFZ	-0.278	0.275	0.038	0.310
BCFZ	0.025	0.298	0.120	0.329
WCFZ	0.021	0.312	0.221	0.369
SCFZ	-0.242	0.248	0.015	0.385
KUFZ	-0.292	0.276	-0.061	0.384

### Spatiotemporal changes in the aridity index (AI) and its correlation with MRSEI

**Fig. 10:**
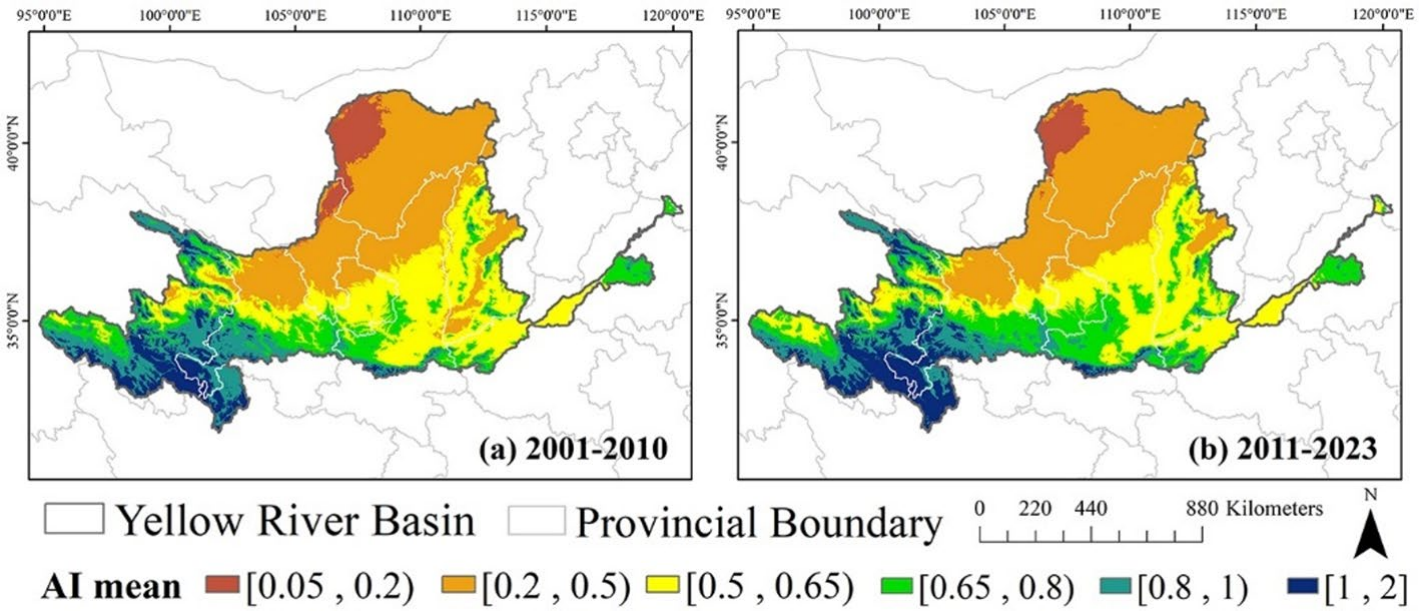
Mean of the Aridity Index from 2001 to 2010 and from 2011to 2023.

For the YRB, the Aridity Index (AI) exhibited a north-to-south increasing trend, reflecting a climatic shift from arid/semi-arid to humid environments (Fig. 10).

**Fig. 11:**
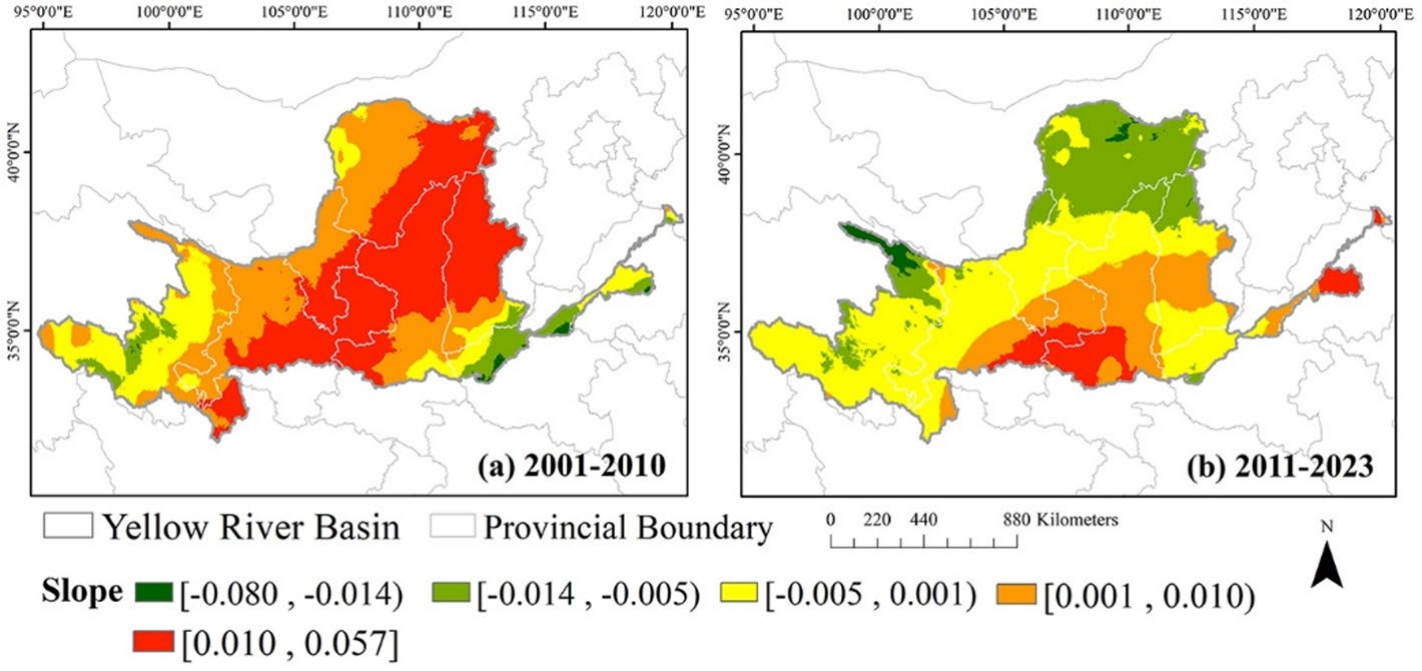
Aridity change trends from 2001 to 2010 and from 2011 to 2023.

For various functional zones, the WSFZ exhibited the lowest average AI values (2001–2010: 0.306 ± 0.107; 2011–2023: 0.330 ± 0.104), indicating a persistently semi-arid climate (Fig. 10). In contrast, the Biodiversity Conservation Functional Zone (BCFZ) and WCFZ recorded relatively higher average AI values (average AI values ≥ 0.849 ± 0.229), indicating a humid environment. As depicted in Fig. 11, from 2001 to 2010, AI in the APFZ and SCFZ displayed increasing trends (0.015 ± 0.006/10a and 0.014 ± 0.008/10a, respectively), while the FRFZ was the only zone showing a decreasing trend (-0.008 ± 0.005/10a). During 2011–2023, decreasing AI trends were observed in the WSFZ and WCFZ (-0.006 ± 0.003/10a, -0.002 ± 0.07/10a, respectively).

Based on the above analysis, it indicated that from 2001 to 2010, the middle reaches of the YRB exhibited a positive AI trend, consistent with the MRSEI trend, mainly also in the SCFZ. Additionally, the correlation between AI and MRSEI was also conducted like temperature.

**Fig. 12:**
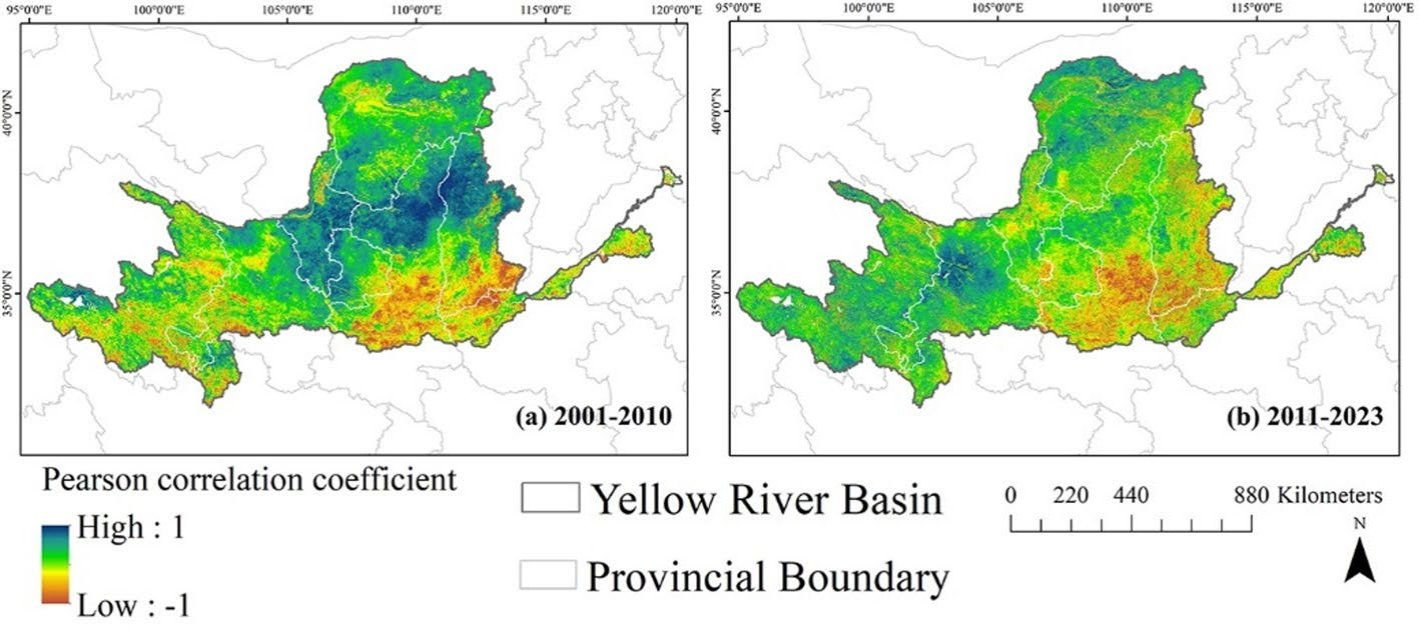
Pearson correlation coefficient between aridity index and MRSEI.

In both periods (2001–2010 and 2011–2023), negative correlations between AI and MRSEI were observed in the southwestern part of the middle reaches, while positive correlations dominated the central and northern parts of the upper and middle reaches, mainly concentrated in the WSFZ (r_2001-2010_=0.520 ± 0.225, r_2011-2023_=0.391 ± 0.270, *p* < 0.01) and the SCFZ (r_2001-2010_=0.467 ± 0.350, r_2011-2023_=0.237 ± 0.299, *p* < 0.01) (Fig. 12; Table 7). These results suggest that the correlation between the AI and MRSEI was stronger in the WSFZ and SCFZ from 2001 to 2010.

**Table 7:** Pearson correlation coefficient between aridity index and MRSEI in different functional zones.

Functional zones	2001–2010		2011–2023	
	Mean	Standard deviation	Mean	Standard deviation
WSFZ	0.520	0.225	0.391	0.270
FRFZ	0.116	0.252	0.099	0.210
FPFZ	0.259	0.195	0.445	0.259
APFZ	0.107	0.341	0.102	0.326
BCFZ	0.049	0.313	0.156	0.330
WCFZ	0.219	0.323	0.349	0.294
SCFZ	0.467	0.350	0.237	0.299
KUFZ	0.191	0.325	0.336	0.444

### Contribution analysis of ecological factors based on linear models

To address the complex interplay between the natural environment and anthropogenic activities in the Yellow River Basin, the R-programs were developed to analyze the driving mechanisms of ecological factors influencing vegetation dynamics. Using the RSEI as the dependent variable, the model incorporates environmental factors—Evapotranspiration (ET), Land Surface Temperature (LST), ALBEDO, Temperature (T), and Aridity Index (AI)—alongside anthropogenic factors, specifically Normal Difference Built-up Index (NDBI), Population Density (POP) and Nighttime Light (NLS) —ecosystem function factors, including Normalized Difference Moisture Index (NDMI), Net Primary Productivity (NPP). By comparing linear (Multiple Linear Regression) and non-linear (Random Forest) models, we quantitatively evaluated the contribution rates of individual variables to the spatial differentiation of vegetation.

#### Construction and evaluation of the multiple linear regression model

Multiple Linear Regression (MLR) was employed to analyze the contributions of various ecological factors to vegetation dynamics. Statistical results were derived from over 200 sampling points randomly extracted via the R-program. The Model performance metrics (Table 8) indicate high explanatory power:

2001–2010: The MLR model achieved a coefficient of determination (R^2^) of 0.987 and an RMSE of 0.032, indicating that the selected indicators provide an excellent explanation of RSEI variations. 2011–2023: The model maintained high consistency, with an R^2^ of 0.986.

#### Analysis of key driving factors

(1) **Dominance of Surface Moisture**: The Normalized Difference Moisture Index (NDMI) contributed 52.75% (2001–2010) and 56.06% (2011–2023) to RSEI, remaining highly significant throughout both periods. This underscores that surface moisture conditions serve as the “primary driving force” for vegetation dynamics in the northern of the Yellow River Basin.

(2) **Localized Effects of Land Exposure**: The contribution of the Normalized Difference Built-up Index (NDBI) ranged from 36.6% to 41.7%, yet these values were not statistically significant. This suggests that while urbanization and land exposure influence RSEI macroscopically, their effects are highly localized at the pixel scale.

(3) **Resurgence of Natural Drivers**: The contribution of Aridity Index (AI) rose from 0.63% to 0.90%, with its significance shifting from “non-significant” to “significant”. As ecological restoration enters a consolidation phase, the influence of natural precipitation is resurging.

(4) **Radiative Feedback Loops**: The weight of Albedo (ALB) more than doubled, from 1.46% to 3.20%. Increased vegetation cover reduces surface albedo, modifying the surface radiative balance and creating a positive feedback loop.

**Table 8:** Factor contributions to vegetation dynamics.

Variables	2001–2010yr	2011–2023yr	Trend / Significance Change
NDMI	52.75%	56.06%	Consistently significant; increasing influence
NDBI	41.74%	36.69%	Consistently non-significant
ALB	1.46%	3.20%	Significant; influence doubled
AI	0.63%	0.90%	Shifted from non-significant to significant
ET	1.08%	0.83%	Consistently significant
NPP	0.82%	0.70%	Shifted from significant to non-significant

Note: Other factors were included in the statistical analysis but are omitted due to negligible values.

### Importance analysis based on random forest

#### Significance of non-linear model

Given the non-linear relationship between vegetation growth and ecological factors, using the Random Forest (RF) model coupled with the DALEX package, SHAP (SHapley Additive exPlanations) values were employed to isolate the pure contribution of ecological factors for the importance analysis based on the dataset form 2001–2023.

#### Importance Ranking Based on SHAP

**Fig. 13:**
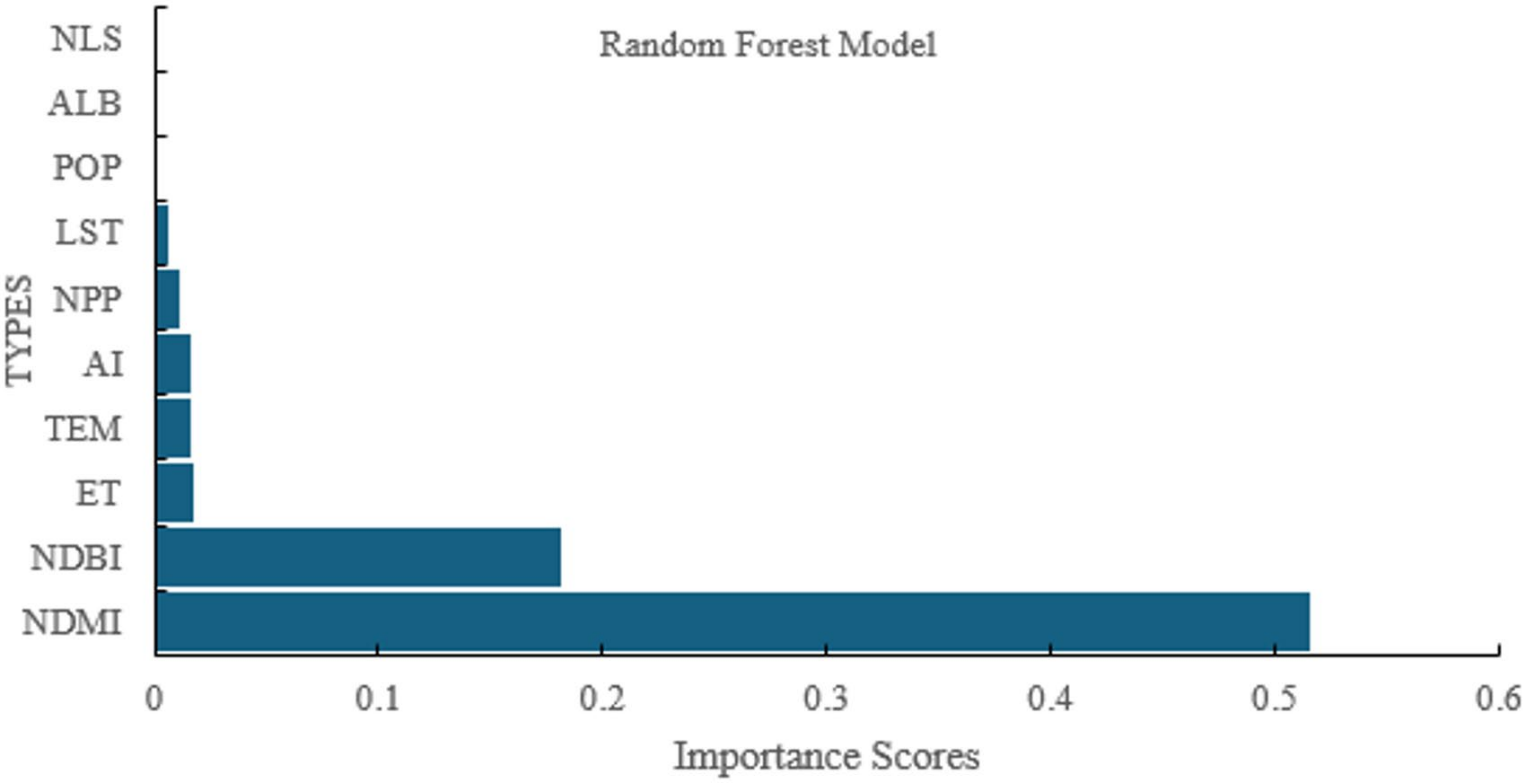
Random forest variable importance, form 2001–2023.

Permutation Importance testing ranked the explanatory power of variables as follows (Fig. 13): NDMI > NDBI > ET > TEM > AI > NPP > LST > POP.

(1) **SHAP Analysis**: NDMI importance scores remained stable between 0.51 and 0.57, significantly outperforming other factors form 2001–2023. This further confirms that “water” is the decisive component within the “hydro-thermal” conditions of this region.

(2) **Non-linear Characteristics of Resource Utilization**: NDBI, as anthropogenic factors, performed more significant in the non-linear model than in the linear model, indicating a threshold effect of NDBI on vegetation rather than a simple linear correlation.

As a morphological indicator of terrestrial vegetation change, the significant growth of RSEI has become one of the more notable changing characteristics over the past two decades. However, the driving mechanism research reveals that growth of RSEI is fundamentally reshaped by ecosystem functional indicators, specifically NDMI and ET. As RSEI increases, the reduction in the percentage of land-cover (such as barred lands) alters the surface energy budget.

## Discussion

### Principal component analysis before and after RSEI model improvement

#### Comparative analysis of different schemes

Ecological quality refers to a comprehensive quantitative evaluation of surface vegetation, water bodies, soil, and temperature. A improved remote sensing ecological index (MRSEI), incorporating greenness, humidity, surface reflectance, and surface temperature, was employed to develop an ecological quality assessment scheme for natural land types—referred to as Plan-1, which was used for assessing ecological quality in the Yellow River Basin, with a comparative analysis against the conventional assessment scheme—Plan-2. The contribution rates of individual indicators are listed in Table 9.

**Table 9:** The correlation matrix between MRSEI and ecological indicators.

Plan-1	NDVI	MNDWI	Albedo	LST	MRSEI
NDVI		0.352	-0.553	-0.746	**0.948**
MNDWI			-0.655	-0.637	0.568
Albedo				0.807	-0.749
LST					**-0.903**
Plan-2	NDVI	NDWI	NDBSI	LST	RSEI
NDVI		-0.745	-0.634	-0.816	0.975
NDWI			0.295	0.446	-0.709
NDBSI				0.715	-0.779
LST					-0.866

Both Plan-1 and Plan-2 consist of four key indicators: water bodies, soil, temperature, and vegetation. However, differences exist in the methods used to obtain these indicators. Ultimately, the information content of RSEI under Plan-1 and Plan-2 was calculated as 79.27% and 78.89%, respectively. Plan-1 demonstrates a slightly higher capacity to capture the information content of the selected indicators. Furthermore, the eigenvalues of the first principal component for Plan-1 and Plan-2 are 0.044 and 0.016, respectively. Given that the first principal component can explain a larger proportion of the variance in the original data, Plan-1 is considered more effective than Plan-2.

In Plan-1, two tasseled cap components correlate positively with the Modified Remote Sensing Ecological Index (MRSEI) (0.948 and 0.568, respectively), reflecting positive contributions of vegetation and water to ecological quality; whereas surface reflectance and temperature exhibited negative effects. In contrast, Plan-2 misrepresents water’s influence on ecological quality.

#### Location-based surface eco-environment comparison

Given that the surface vegetation types in the Yellow River Delta are complex and significantly affected by anthropogenic fluctuations, the evaluation results of ecological quality on its terrestrial surface were selected for confirmatory analysis. Furthermore, the comparison and analysis were conducted for the years 2018 and 2023 (the years when UAV data sampling was performed).

Plan1 demonstrated superior recognition accuracy for saline-alkali and barren lands compared with Plan2, while its ecological quality estimates shown in Figs. 15, 16 − 2,3,4 were lower than those of Plan2, aligning more closely with the UAV-based validation results. To further validate the accuracy of the two schemes, random sample points were selected within areas where MRSEI values ranged from 0 to 0.5 for comparative analysis. The R^2^ values of Plan1 was 0.2018, significantly higher than that of Plan2 (0.0275), and its slope was approximately 1, indicating that Plan2 has stronger consistency and reliability in ecological quality evaluation in the low ecological quality range. Overall, Plan1 demonstrated the best performance.

**Fig. 14:**
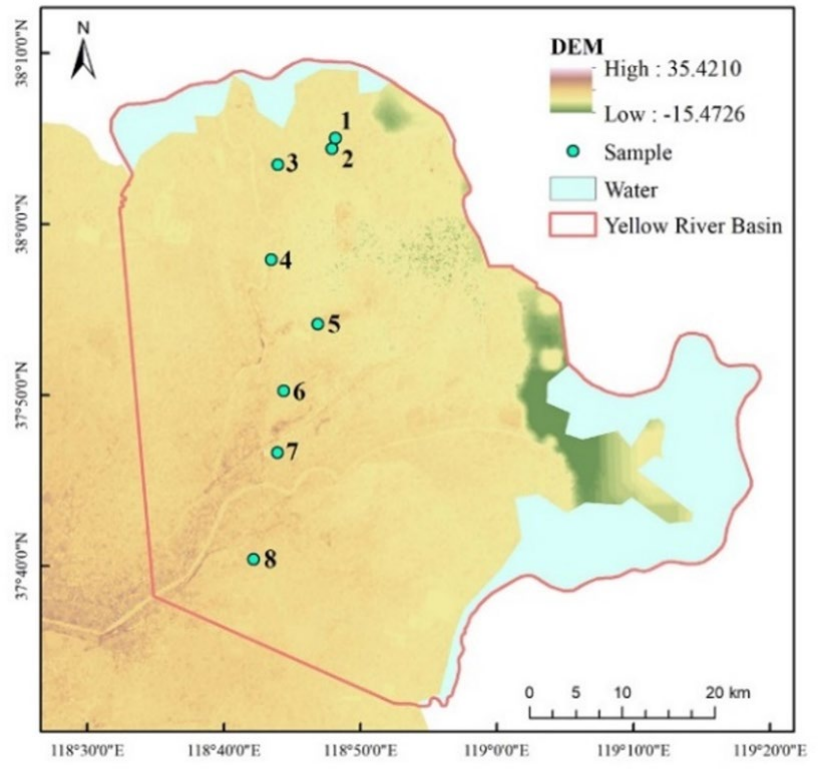
Field sampling sites based on USA.

**Fig. 15:**
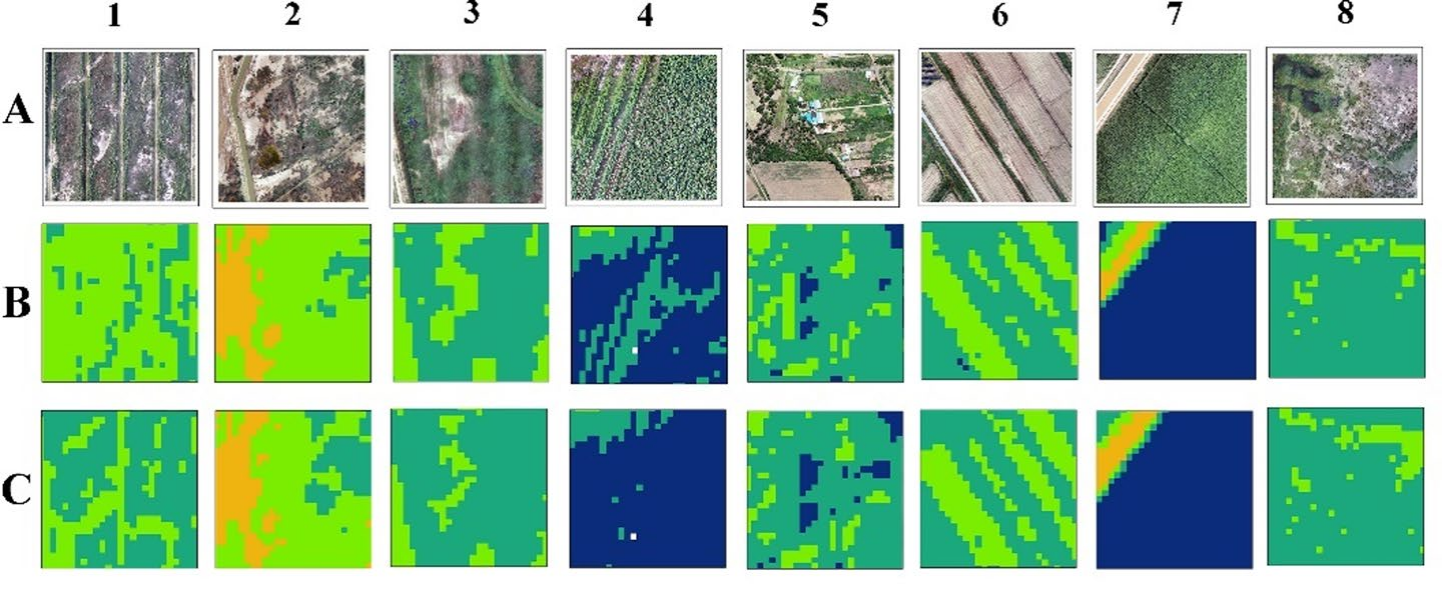
Comparison between UAV-observed plots (2023.7) and scheme evaluation results (A: field plots; B-C show the ecological quality evaluation results of Plan1and Plan2, respectively, and 1–8 correspond to the eight sampling sites shown in Fig. 14.)

**Fig. 16:**
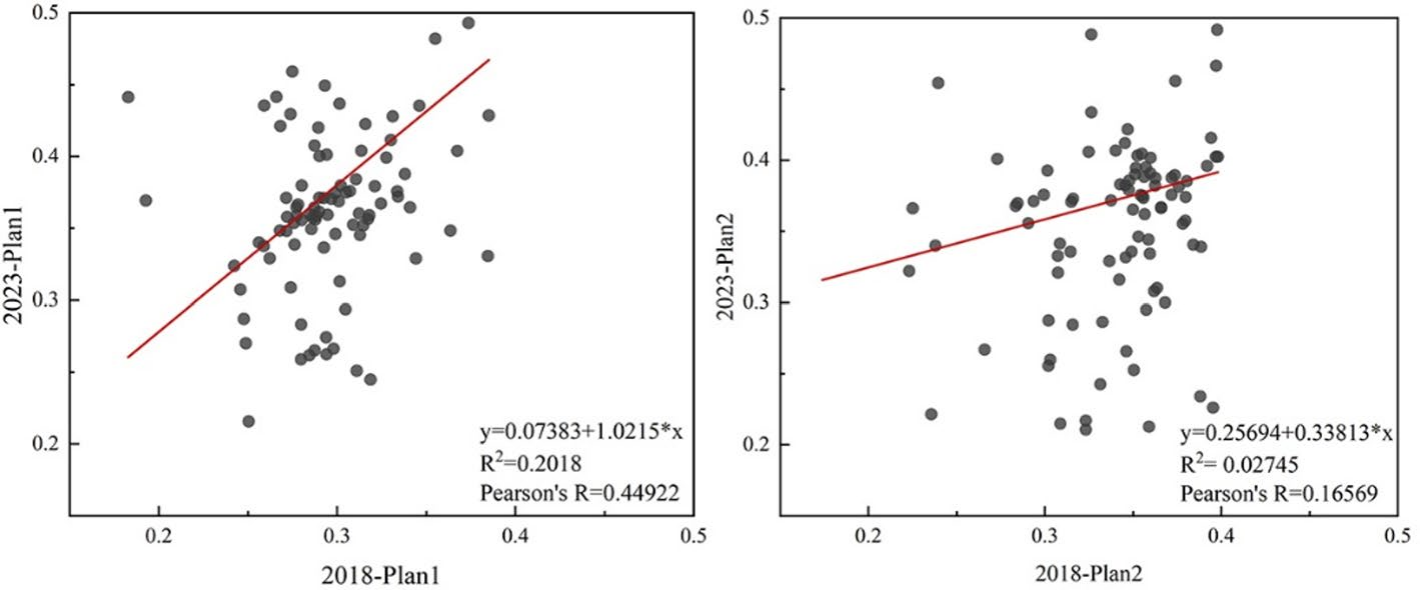
Correlation of ecological quality evaluation results in the low ecological quality range.

### The variation levels of terrestrial ecological quality

Against the backdrop of China’s ecological restoration efforts, terrestrial ecological changes have exhibited distinct regional patterns, as reflected by vegetation indices (Normalized Difference Vegetation Index, NDVI) derived from MODIS or Landsat data. Over the past two decades, vegetation coverage growth rates in ecological engineering zones of eastern China has averaged approximately 5%-15% per decade^39^. Within the study area, absolute ecological change rates across various land-cover types range from 10.3% to 20.13% per decade, aligning closely with this regional trend. In other GFPP (the Grain-for-Green Program, GFPP)-implemented regions, annual vegetation coverage growth averages 1%-3%, with cumulative increases reaching 10%-30% per decade^40^. However, the Yellow River Basin exhibits unique dynamics: under effective governance, the middle-lower basin’s forest land has shown a cumulative annual dynamic change rate of 13.87% per decade.

Driven by human intervention, the GFPP has significantly boosted northern China’s vegetation coverage, with a cumulative growth rate of 20% per decade^41^. Specifically, in the North/Northwest China’s cropland-to-forest/grassland conversion zones under the GFPP, NDVI annual growth ranges from 1.5% to 2.5%, with cumulative increases of 12%-27% per decade^42^. The Loess Plateau core area has seen a 25%-30% cumulative vegetation coverage increase, accompanied by NDVI dynamics of 1.5%-3% annually^43^. In Southwest China’s Hengduan Mountains, post-GFPP vegetation coverage grows at 1.2%-2.8% annually, cumulatively increasing by 15%-25% per decade^44^.

Most existing studies rely on MODIS NDVI (characterized by lower spatial resolution but strong temporal continuity) or Landsat NDVI (with high spatial resolution but susceptibility to cloud interference), potentially causing growth rate discrepancies. High-temporal-resolution change detection algorithms mitigate these differences by reducing noise and atmospheric interference^45^. Collectively, northern China—including the middle-lower Yellow River region—falls within the moderate-low ecological growth zone. Natural land covers (grasslands, bare lands) exhibit dynamic variation rates of 18.676% and 19.213% per decade, respectively.

### Relationship between land cover conversion and terrestrial ecological quality

**Fig. 17:**
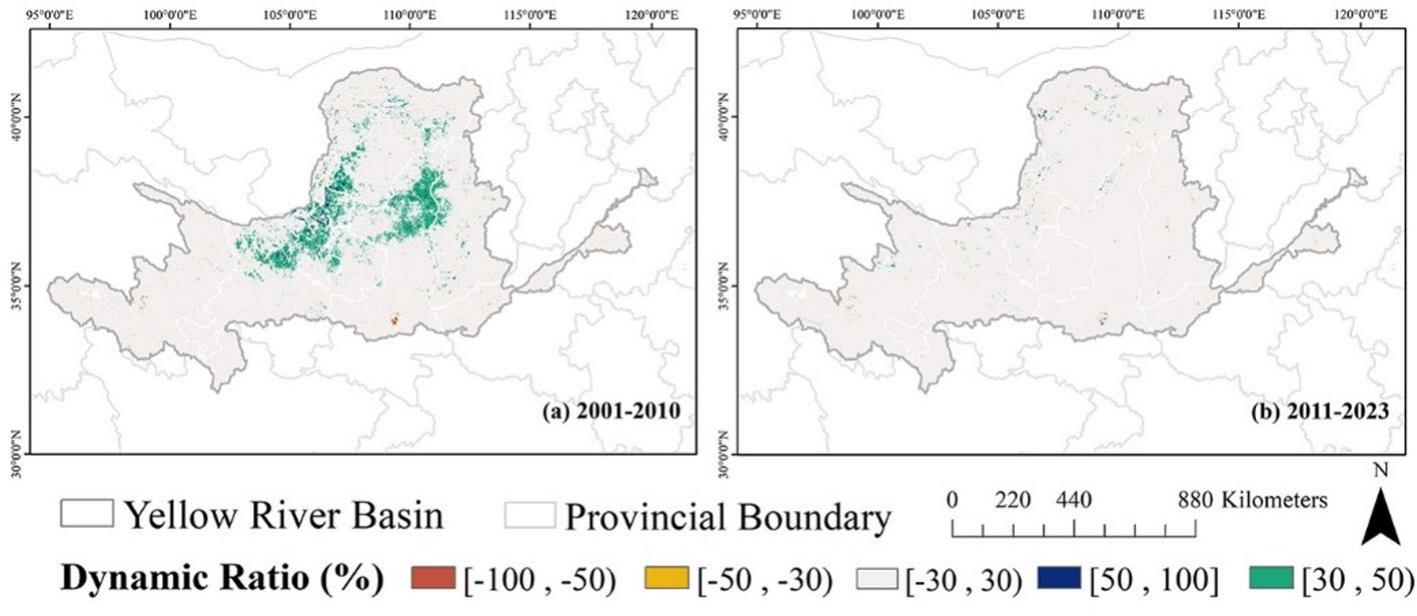
Dynamic ratio detection. Note: Ecological quality changes were classified into five levels: significant degradation [-100, -50), moderate degradation [-50, -30), stable [-30, 30), moderate improvement [30, 50), and significant improvement [50, 100].

Based on the annual MRSEIs, the dynamic ratio was calculated, and two intervals —^24,46^ and [50, 100] —were selected to indicate the ecological improvement ranges, considering the actual circumstances of the YRB. By extracting the areas of land cover conversion within these two levels (Fig. 17), we found that from 2001 to 2010, land cover conversion resulted from eco-variation accounted for 13.566% and 24.218% of the total conversion area, respectively; from 2011 to 2023, these proportions increased to 27.155% and 46.184% (Table 9). Notably, although the areas of the improved ecological quality from 2001 to 2010 (59,728.938 km^2^) are larger than that from 2011 to 2023 (3,129.750 km^2^), the proportion of land cover conversion was lower in 2001–2010 (14.20%) than in 2011–2023 (35.70%). This suggests that land cover conversion was not the main factors for the improvement of ecological quality in the YRB from 2001 to 2010.

Specifically, in regions where ecological conditions improved, the dominant land cover conversion types from 2001 to 2023 included barren land -to-cropland conversion (3,640.44 km²) and cropland/barren land-to-grassland conversion (3,212.19 km²; 1,507.25 km²). These were followed by conversions to impervious surfaces and forests. For the relationship between land use types and MRSEI, Yu et al. and Ma et al. have also conducted relevant research: Yu et al. considered that grassland expansion of Jinan (lower YRB) played a key role in ecological improvement, whereas urban expansion contributed to ecological deterioration^47^; Ma et al., however, concluded that there was no significant correlation between forest and MRSEI in the Yellow River Delta^48^. These findings align with the conclusions drawn in this paper. However, Yu et al. also reported that the transfer of grassland/woodland to farmland exacerbated ecological degradation^47^. This discrepancy may be attributed to differences in regional heterogeneity. Unlike Jinan, the YRB spans nine provinces, each characterized by their natural conditions, economic development levels, and policy implementation strategies. Furthermore, the observed expansion of cropland in improved regions likely reflects the implementation of high-standard farmland and irrigation projects which, while increasing MRSEI through higher moisture and greenness, must be distinguished from natural ecosystem restoration.

**Table 9:** Land cover types conversion in regions with improved ecological quality.

Dynamic Ratio (%)	2001–2010		2011–2023	
	[30,50)	[50,100]	[30,50)	[50,100]
cropland to grassland	5.451	2.520	2.206	1.010
barren land to cropland	4.732	12.317	15.262	25.153
barren land to grassland	2.033	6.110	4.370	5.963
Total areas* (km^2^)	13.566	24.218	27.155	46.184

Note: Table 9 showed the main land cover types conversion (area >1km^2^); and the Total areas* indicated the total area of all land cover types conversion, including area≤1km^2^.

### Recommendations for functional zones based on terrestrial ecological quality

The YRB is divided into eight functional zones, with the WSFZ, WCFZ, SCFZ and FRFZ as representative functional zones reflecting ecological quality status in the upper, middle, and lower reaches of the YRB.

Analysis in this study revealed that areas classified as Poor and Fair were concentrated in the WSFZ and SCFZ in the upper and middle reaches of the YRB, consistent with Yang et al.^49^, who considered unreasonable human activities as the main reason to explain the phenomenon. For the WSFZ and SCFZ, belonging to semi-arid climate and exhibiting stronger correlation with AI, it is recommended to enhance ecological quality and climate adaptability through multi-dimensional coordination strategies. These strategies may include: (1) optimizing water resource management and land restoration practices (e.g., adjusting planting structures and irrigation methods to conserve water^50^; (2) strengthening soil stabilization via ecological engineering techniques; and (3) implementing long-term vegetation dynamics monitoring^51^.

For the WCFZ (upper reaches) and the FRFZ (lower reaches), the levels of RSEI belong to Good, but they have no significant correlation with temperature and AI. It is essential to maintain the current ecological quality status in these two functional zones. Specific measures include: (1) prioritizing ecological monitoring and implement ecological protection policies for the WCFZ^52^; and (2) strengthening land-use zoning regulations in rapidly expanding cities (e.g., Zhengzhou) to prevent ecological degradation in the FRFZ^46^.

## Conclusions

This study investigates the spatiotemporal dynamics of ecological quality in the Yellow River Basin (YRB) from 2001 to 2023 using the Modified Remote Sensing Ecological Index (MRSEI), examines the correlations between MRSEI and climatic factors (such as the temperature utilization efficiency index and Aridity Index), and explores the relationship between land cover conversion and ecological quality improvement. The main conclusions of this paper follow:

Firstly, ecological quality in the YRB remained relatively stable overall, with significant improvements observed in the Soil Conservation Functional Zone (SCFZ) of the middle reaches between 2001 and 2010. Furthermore, the primary land cover conversion types in regions with ecological quality improvement were the expansion of cropland and grassland. Secondly, a significant correlation between ecological quality (MRSEI) and the Aridity Index (AI) was observed in the Windbreak and Sand Fixation Zone (WSFZ) and Soil Conservation Functional Zone (SCFZ) across the upper and middle reaches during 2001–2010. The moisture index (NDMI) emerged as the primary driver (> 50% contribution), confirming that the water cycle budget—rather than thermal variability—governs ecological quality in this arid-to-semi-arid basin. Meanwhile, we have also quantified the extent of ecological impacts caused by land use/cover conversions induced by anthropogenic activities within areas experiencing significant ecological shifts. Notably, anthropogenic activities have not been predominant drivers of ecological changes over the last decade. These findings offer critical insights for guiding ecological conservation and sustainable development in the functional zones of the YRB.

## Data Availability

Ye, H., and Li, X. (2025). Dataset of the yearly Eco-Environmental Quality in the Yellow River Basin [Dataset]. Zenodo. https://doi.org/10.5281/zenodo.16872271 .

## References

[1] Men, D. & Pan, J. Incorporating network topology and ecosystem services into the optimization of ecological network: A case study of the Yellow River Basin. *Sci. Total Environ.* **912**, 169004 (2024). 38040351 10.1016/j.scitotenv.2023.169004

[2] Li, C. et al. Comprehensive evaluation of the high-quality development of the ecological and economic belt along the Yellow River in Ningxia. *Sustainability* **15** (15), 11486 (2023).

[3] Zhang, B. et al. Construction of watershed ecological security patterns with integrated of spatial variability: A case study of the Yellow River Basin, China. *Ecol. Ind.* **159**, 111663 (2024).

[4] Feng, L. et al. Effects of mosaic biological soil crusts on vascular plant establishment in a coastal saline land of the Yellow River Delta, China. *J. Plant Ecol.* **14**(5), 781–792 (2021).

[5] Guo, S. et al. Threshold effect of ecosystem services in response to climate change, human activity and landscape pattern in the upper and middle Yellow River of China. *Ecol. Ind.* **136**, 108603 (2022).

[6] Cui, Q., Xia, J., Yang, H., Liu, J. & Shao, P. Biochar and effective microorganisms promote Sesbania cannabina growth and soil quality in the coastal saline-alkali soil of the Yellow River Delta, China. *Sci. Total Environ.* **756**, 143801 (2021). 10.1016/j.scitotenv.2020.143801 33307496

[7] Wu, H., Shi, P., Qu, S., Zhang, H. & Ye, T. Establishment of watershed ecological water requirements framework: A case study of the Lower Yellow River, China. *Sci. Total Environ.* **820**, 153205 (2022). 35063531 10.1016/j.scitotenv.2022.153205

[8] Wang, S. et al. Identification and optimization of ecological corridors in the middle reaches of the Yellow River Basin. *J. Clean. Prod.* 145676. (2025).

[9] Ying, X. et al. Combining AHP with GIS in synthetic evaluation of eco-environment quality-A case study of Hunan Province, China. *Ecol. Model.* **209** (2–4), 97–109 (2007).

[10] Romero-Calcerrada, R. & Luque, S. Habitat quality assessment using Weights-of-Evidence based GIS modelling: The case of Picoides tridactylus as species indicator of the biodiversity value of the Finnish forest. *Ecol. Model.* **196** (1–2), 62–76 (2006).

[11] Yu, S. et al. Measurement of land ecological security in the middle and lower reaches of the Yangtze River Base on the PSR Model. *Sustainability* **15** (19), 14098 (2023).

[12] Ge, J. et al. IRSEI-based monitoring of ecological quality and analysis of drivers in the Daling River Basin. *Sci. Rep.* **14** (1), 14506 (2024). 38914680 10.1038/s41598-024-65511-5 PMC11196709

[13] Yan, Z., Zhou, Z., Liu, J., Wang, H. & Li, D. Water use characteristics and impact factors in the Yellow River basin, China. *Water Int.* **45** (3), 148–168 (2020).

[14] Wang, Y., Kong, X., Guo, K., Zhao, C. & Zhao, J. Spatiotemporal change in vegetation cover in the Yellow River Basin between 2000 and 2022 and driving forces analysis. *Front. Ecol. Evol.* **11**, 1261210 (2023).

[15] Ren, Z., Tian, Z., Wei, H., Liu, Y. & Yu, Y. Spatiotemporal evolution and driving mechanisms of vegetation in the Yellow River Basin, China during 2000–2020. *Ecol. Ind.* **138**, 108832 (2022).

[16] Wiegand, T., Naves, J., Garbulsky, M. F. & Fernández, N. Animal habitat quality and ecosystem functioning: exploring seasonal patterns using NDVI. *Ecol. Monogr.* **78** (1), 87–103 (2008).

[17] Xu, H., Wang, Y., Guan, H., Shi, T. & Hu, X. Detecting ecological changes with a remote sensing based ecological index (RSEI) produced time series and change vector analysis. *Remote Sens.* **11** (20), 2345 (2019).

[18] Xu, H. Q. A remote sensing urban ecological index and its application. *Acta Ecol. Sin*. **33** (24), 7853–7862 (2010).

[19] Geng, J. et al. Analysis of spatiotemporal variation and drivers of ecological quality in Fuzhou based on RSEI. *Remote Sens.* **14** (19), 4900 (2022).

[20] Zhou, J. & Liu, W. Monitoring and evaluation of eco-environment quality based on remote sensing-based ecological index (RSEI) in Taihu Lake Basin, China. *Sustainability* **14** (9), 5642 (2022).

[21] Zheng, Z., Wu, Z., Chen, Y., Guo, C. & Marinello, F. Instability of remote sensing based ecological index (RSEI) and its improvement for time series analysis. *Sci. Total Environ.* **814**, 152595 (2022). 34995601 10.1016/j.scitotenv.2021.152595

[22] Liu, Y., Xiang, W., Hu, P., Gao, P. & Zhang, A. Evaluation of ecological environment quality using an improved remote sensing ecological index model. *Remote Sens.* **16** (18), 3485 (2024).

[23] Wang, J., Jiang, L., Qi, Q. & Wang, Y. An ecological quality evaluation of large-scale farms based on an improved remote sensing ecological index. *Remote Sens.* **16** (4), 684 (2024).

[24] Chen, N., Cheng, G., Yang, J., Ding, H. & He, S. Evaluation of urban ecological environment quality based on improved RSEI and driving factors analysis. *Sustainability* **15** (11), 8464 (2023).

[25] Xu, C. et al. Effects of land use/cover change on carbon storage between 2000 and 2040 in the Yellow River Basin, China. *Ecol. Ind.* **151**, 110345 (2023).

[26] Wang, Y., Song, J., Li, Q. & Jiang, X. Exploration of the development of water-energy-food nexus and its endogenous and exogenous drivers in the Yellow River Basin, China. *J. Environ. Manage.* **378**, 124735 (2025). 40037254 10.1016/j.jenvman.2025.124735

[27] Yang, H. et al. Effects of different Tamarix chinensis-grass patterns on the soil quality of coastal saline soil in the Yellow River Delta, China. *Sci. Total Environ.* **772**, 145501 (2021). 10.1016/j.scitotenv.2021.145501 33571770

[28] Du, L., Dong, C., Kang, X., Qian, X. & Gu, L. Spatiotemporal evolution of land cover changes and landscape ecological risk assessment in the Yellow River Basin, 2015–2020. *J. Environ. Manage.* **332**, 117149 (2023). 36808004 10.1016/j.jenvman.2022.117149

[29] Zhao, J. et al. Carbon emission prediction model and analysis in the Yellow River basin based on a machine learning method. *Sustainability* **14** (10), 6153 (2022).

[30] Peng, S. *1-km monthly precipitation dataset for China (1901–2022)* (Beijing, China, 2020).

[31] Peng, S. *1-km monthly potential evapotranspiration dataset for China (1901–2023) National Tibetan* (Plateau/Third Pole Environment Data Center [data set], 2022).

[32] Yang, J. & Huang, X. 30 m annual land cover and its dynamics in China from 1990 to 2019. *Earth Syst. Sci. Data Discuss.* 1–29. (2021).

[33] Zhang, Y. et al. Assessment of coastal ecological restoration effectiveness using an improved remote sensing ecological index: a case study of the Liaohe Estuary. *Front. Ecol. Evol.* **13**, 1603614 (2025).

[34] Sahin, S. An aridity index defined by precipitation and specific humidity. *J. Hydrol.* **444**, 199–208 (2012).

[35] Bolker, B. M. et al. Generalized linear mixed models: a practical guide for ecology and evolution. *Trends Ecol. Evol.* **24** (3), 127–135 (2009). 19185386 10.1016/j.tree.2008.10.008

[36] Strobl, C., Malley, J. & Tutz, G. An introduction to recursive partitioning: Rationale, application, and characteristics of classification and regression trees, bagging, and random forests. *Psychol. Methods*. **14** (4), 323–348 (2009). 19968396 10.1037/a0016973 PMC2927982

[37] Yang, W., Zhou, Y. & Li, C. Assessment of ecological environment quality in rare earth mining areas based on improved RSEI. *Sustainability* **15** (4), 2964 (2023).

[38] Wen, C., Long, T., He, G., Jiao, W. & Jiang, W. Temporally enhanced RSEI and nighttime lights reveal long-term ecological changes and effective protection in China’s inaugural national parks. *Ecol. Ind.* **170**, 112981 (2025).

[39] Wu, Z. et al. Satellite-based large-scale vegetation dynamics in ecological restoration programs of Northern China[J]. *Int. J. Remote Sens.* **40** (5–6), 2296–2312 (2019).

[40] Yin, R. S., Yin, G. P. & Li, L. Y. Assessing China’s Ecological Restoration Programs: What’s Been Done and What Remains to Be Done? [J]. *Environ. Manage.* **45** (3), 442–453 (2010). 19847479 10.1007/s00267-009-9387-4

[41] Niu, Q. F. et al. *Ecological engineering projects increased vegetation cover, production, and biomass in semiarid and subhumid Northern China* Vol. 30, 1620–1631 (Land Degradation & Development[J], 2019). 13.

[42] Qiao, X. et al. Spatiotemporal Changes in Vegetation Cover during the Growing Season and Its Implications for Chinese Grain for Green Program in the Luo River Basin[J]. *Forests* **15** (9), 1649–1649 (2024).

[43] Cao, S. X., Chen, L. & Yu, X. X. Impact of China’s Grain for Green Project on the landscape of vulnerable arid and semi-arid agricultural regions: a case study in northern Shaanxi Province[J]. *J. Appl. Ecol.* **46** (3), 536–543 (2009).

[44] Fan, M. & Xiao, Y. T. *Impacts of the grain for Green Program on the spatial pattern of land uses and ecosystem services in mountainous settlements in southwest China[J]* 21 (Global Ecology and Conservation, 2020).

[45] Kennedy, R. E., Yang, Z. Q. & Cohen, W. B. Detecting trends in forest disturbance and recovery using yearly Landsat time series: 1. LandTrendr—A new algorithm[J]. *Remote Sens. Environ.* **114** (12), 2897–2910 (2010).

[46] Wang, Y. et al. Coupling and Coordination Analysis of Land Use Function and Ecological Quality in Yellow River Basin, Henan Province, China. *Sustainability* **16** (23), 10699 (2024).

[47] Yu, G. et al. Impact of land use/land cover change on ecological quality during urbanization in the lower Yellow River Basin: A case study of Jinan City. *Remote Sens.* **14** (24), 6273 (2022).

[48] Ma, D., Huang, Q., Liu, B. & Zhang, Q. Analysis and dynamic evaluation of Eco-environmental Quality in the Yellow River Delta from 2000 to 2020. *Sustainability* **15** (10), 7835 (2023).

[49] Yang, Z. et al. Analysis of ecological environmental quality change in the Yellow River Basin using the remote-sensing-based ecological index. *Sustainability* **14** (17), 10726 (2022).

[50] Zhang, X., Wang, G., Xue, B., Wang, Y. & Wang, L. Spatiotemporal variation of evapotranspiration on different land use/cover in the inner Mongolia reach of the Yellow River Basin. *Remote Sens.* **14** (18), 4499 (2022).

[51] Zhang, X., Wang, G. & Xue, B. Changes in vegetation cover and its influencing factors in the inner Mongolia reach of the yellow river basin from 2001 to 2018. *Environ. Res.* **215**, 114253 (2022). 36067843 10.1016/j.envres.2022.114253

[52] Shen, Z. & Gong, J. Spatial–Temporal Changes and Driving Mechanisms of Ecological Environmental Quality in the Qinghai–Tibet Plateau, China. *Land* **13** (12), 2203 (2024).

